# A family case of a rare Xq28 duplication

**DOI:** 10.18699/vjgb-25-69

**Published:** 2025-09

**Authors:** A.E. Kopytova, E.N. Tolmacheva, D.A. Emelina, O.S. Glotov, V.V. Miroshnikova, T.S. Usenko, O.Yu. Vasilyeva, I.V. Makarov, A.D. Lobanov, G.E. Mazo, S.N. Pchelina, I.N. Lebedev

**Affiliations:** Petersburg Nuclear Physics Institute named by B.P. Konstantinov of National Research Center “Kurchatov Institute”, Gatchina, Leningradskaya Oblast, Russia Pavlov First St. Petersburg State Medical University, St. Petersburg, Russia; Scientific Research Institute of Medical Genetics, Tomsk National Research Medical Center of the Russian Academy of Sciences, Tomsk, Russia; V.M. Bekhterev National Research Medical Center for Psychiatry and Neurology of the Ministry of Health of the Russian Federation, St. Petersburg, Russia; Federal Scientific and Clinical Center of Infectious Diseases of the Federal Medical and Biological Agency, St. Petersburg, Russia; Petersburg Nuclear Physics Institute named by B.P. Konstantinov of National Research Center “Kurchatov Institute”, Gatchina, Leningradskaya Oblast, Russia Pavlov First St. Petersburg State Medical University, St. Petersburg, Russia; Petersburg Nuclear Physics Institute named by B.P. Konstantinov of National Research Center “Kurchatov Institute”, Gatchina, Leningradskaya Oblast, Russia Pavlov First St. Petersburg State Medical University, St. Petersburg, Russia; Scientific Research Institute of Medical Genetics, Tomsk National Research Medical Center of the Russian Academy of Sciences, Tomsk, Russia; V.M. Bekhterev National Research Medical Center for Psychiatry and Neurology of the Ministry of Health of the Russian Federation, St. Petersburg, Russia North-Western State Medical University named after I.I. Mechnikov, St. Petersburg, Russia; Siberian State Medical University, Tomsk, Russia; V.M. Bekhterev National Research Medical Center for Psychiatry and Neurology of the Ministry of Health of the Russian Federation, St. Petersburg, Russia; Petersburg Nuclear Physics Institute named by B.P. Konstantinov of National Research Center “Kurchatov Institute”, Gatchina, Leningradskaya Oblast, Russia Pavlov First St. Petersburg State Medical University, St. Petersburg, Russia; Scientific Research Institute of Medical Genetics, Tomsk National Research Medical Center of the Russian Academy of Sciences, Tomsk, Russia

**Keywords:** Xq28 duplication syndrome, array-based comparative genomic hybridization, copy number variations (CNVs), intellectual disability, RAB39B, CLIC2For, синдром дупликации Xq28, матричная сравнительная геномная гибридизация, вариации числа копий участков ДНК (CNV), интеллектуальная недостаточность, RAB39B, CLIC2

## Abstract

Genetic factors contribute to the etiology of intellectual disability in 25–50 % of cases. Chromosomal abnormalities, such as microdeletions and microduplications, are the most significant genetic causes. We examined a family where two boys, aged 8 and 7, were diagnosed with mild intellectual disability. Using array-based comparative genomic hybridization, we detected a duplication of Xq28 in both brothers on the X chromosome inherited from a healthy mother with skewed (88 %) X-chromosome inactivation. The size of the rearrangement is 439.6 kilobases (kb). Eight genes are located in this region, including F8, MTCP1, BRCC3, VBP1, RAB39B, CLIC2, FUNDC2, and CMC4. This chromosomal region overlaps with the region of Xq28 duplication syndrome (OMIM 300815), characterized by intellectual disability, behavioral and psychiatric disorders, recurrent infections, atopic diseases, and specific facial features in affected male individuals. Whole-exome sequencing did not reveal pathogenic or likely pathogenic variants associated with neurodevelopmental disorders. These disorders have been previously linked to X-linked recessive single-nucleotide variants in RAB39B (OMIM 300271, 311510) and CLIC2 (OMIM 300886). An assessment of the clinical significance of the identified duplication, using the AutoCNV internet resource and original data, allowed us to classify this variant as pathogenic. This implies that the identified duplication may be the cause of intellectual disability in patients.К настоящему времени известно, что на долю генетических факторов, вносящих вклад в этиоло- гию нарушения интеллектуального развития, приходится от 25 до 50 % случаев. Среди генетических причин наиболее существенную роль играют хромосомные аномалии, в том числе микроделеции и микродупли- кации. Нами обследована семья, в которой у двух мальчиков в возрасте 8 и 7 лет диагностирована легкая интеллектуальная недостаточность. С помощью матричной сравнительной геномной гибридизации у обоих братьев была обнаружена дупликация Xq28. Мать мальчиков является носительницей такой же дупликации с 88 % смещением инактивации Х-хромосомы. Размер перестройки составил 439.6 т. п. н. В данном регионе локализовано восемь генов (F8, MTCP1, BRCC3, VBP1, RAB39B, CLIC2, FUNDC2, CMC4). Рассматриваемый хромо- сомный регион перекрывается с областью синдрома дупликации Xq28 (OMIM 300815), характеризующегося интеллектуальной недостаточностью, поведенческими и психиатрическими нарушениями, рецидивирую- щими инфекциями, атопическими заболеваниями и характерными чертами лица у мужчин. Ранее описаны нарушения интеллектуального развития, обусловленные рецессивными однонуклеотидными вариантами в генах RAB39B (OMIM 300271, OMIM 311510) и CLIC2 (OMIM 300886). Полноэкзомное секвенирование не выяви- ло дополнительных патогенных и потенциально патогенных вариантов, ассоциированных с нарушениями интеллектуального развития. Оценка клинической значимости обнаруженной дупликации с помощью ин- тернет-ресурса AutoCNV и собственных данных позволила классифицировать этот вариант как патогенный, что предполагает, что он может быть причиной интеллектуальной недостаточности у пациентов.

## Introduction

Intellectual disability (ID) is a group of disorders characterized by limitations in both intellectual functioning and adaptive behavior (cognitive, speech, social abilities). According to the World Health Organization (2021), approximately 1–3 % of the population suffers from various forms of ID (Schalock et al., 2021). Genetic causes of ID are thought to be present in 25–50 % of cases (Lavrov et al., 2016).

Recent studies have shown that copy number variations (CNVs) are found in 15–25 % of patients with ID and/or multiple congenital anomalies (Iyer, Girirajan, 2015; Fedotov et al., 2024) and may play an important role in the etiology of ID. CNVs are changes in the number of copies of a specific DNA segment, such as microdeletions and microduplications, ranging from a few thousand base pairs to several megabases (Kearney et al., 2011). Xq28 duplication syndrome (OMIM #300815) is the most common cause of ID in men and has several variants depending on the genes involved and the extent of the duplication (Tolmacheva et al., 2022). The variant associated with increased copies of the region including the RAB39B and CLIC2 genes is rare and has been described only in a few studies (El-Hattab et al., 2011; Vanmarsenille et al., 2014; Ballout et al., 2021). The manifestations of the disease phenotype are speculated to be the result of an increased dosage of two genes located in the duplicated segment: RAB39B and CLIC2. However, the underlying molecular mechanism remains largely unknown and the contribution of excessive RAB39B to the development of ID has yet to be confirmed (Wang Z. et al., 2023). It is necessary to describe new cases associated with increased doses of the RAB39B and CLIC2 genes in order to clarify their role in the etiology of ID.

We presented a clinical case of two male ID patients with a rare Xq28 duplication. The aim of this study is to describe this duplication, which involves candidate genes RAB39B and CLIC2 in order to better understand their effects and potential contribution to the ID phenotype

## Materials and methods

This study was approved by the Ethics Committee of the V.M. Bekhterev National Research Medical Center for Psychiatry and Neurology of the Russian Federation Ministry of Health (Protocol No. 3 dated 04/25/24). Written informed consent was obtained from parents for themselves and their children

Peripheral blood samples from patients and their parents were obtained from the V.M. Bekhterev National Research Medical Center’s Biobank

The peripheral blood of patients and their relatives was collected in tubes containing EDTA for molecular genetic analyses. Genomic DNA was isolated from blood using phenol- chloroform extraction.

Chromosomal microarray analysis (array Comparative Genomic Hybridization (aCGH)) was performed using SurePrint G3 Human CGH 8×60K microarrays (Agilent Technologies, USA) according to the manufacturer’s recommendations. Detection was performed using the SureScan Microarray Scanner (Agilent Technologies, USA). Data were obtained using the Scan software (version 9.1.1.1) and visualized with the Cytogenomics software (version 3.0.6.0). Interpretation of the clinical significance of CNVs was carried out in accordance with the American College of Medical Genetics and Genomics (ACMG), Clinical Genomics Resource project and the Russian Society of Medical Geneticists (Brandt et al., 2020; Riggs et al., 2020; Lebedev et al., 2023), as well as the DGV, OMIM and DECIPHER databases. A detailed analysis of the clinical signs was conducted by reviewing literature data. The pathogenetic significance of the duplication was classified using the AutoCNV score (https://phoenix. bgi.com/autocnv/) and the assessment of the X-chromosome inactivation status (Tolmacheva et al., 2025). CNVs were classified as pathogenic if they had a total score of ≥0.99 according to a semi-quantitative scoring system (Lebedev et al., 2023).

To confirm a detected CNV in patients and determine its origin, we used real-time quantitative PCR with primers selected for exon 3 of the CMC4 gene (F 5ʹ-CTGTCATCC AAGAACTGCGTAA-3ʹ, R 5ʹ-TACTTTGATGCAGACTT CCGTG-3ʹ).

X-chromosome inactivation status was determined based on the amplification of a highly polymorphic CAG repeat in the first exon of the androgen receptor (AR, Xq12) gene after DNA hydrolysis with the methyl-sensitive restriction endonuclease HpaII. PCR products were separated using fragment analysis. The degree of inactivation <80 % was considered a random pattern, and the degree of inactivation >80 % was considered a skewed X-chromosome inactivation (sXCI).

Whole-exome sequencing. The libraries were prepared using the KAPA HyperExome panel (Roche, USA), according to the manufacturer’s protocol. The sequencing of the converted libraries (MGI Easy Universal Library Conversion Kit (App-A), MGI, China) was performed on a DNBSEQ-G50 NGS sequencer (MGI, China). After sequencing, FastQC was used for quality control to assess the raw sequence data (Andrews, 2020). The data obtained from sequencing experiments were aligned to the human reference genome, specifically the GRCh38 assembly, using an algorithm called Burrows– Wheeler Aligner (BWA v.0.7.17) (http://bio-bwa.sourceforge. net/). To eliminate possible duplication artifacts at the amplification stage, we used the GATK MarkDuplicates tool to identify and remove PCR duplicates (McKenna et al., 2010). After initial read mapping, the next steps involved recalibrating the quality scores of the reads and addressing potential biases in short insertion/deletion calls. This was achieved using the GATK Base Quality Score Recalibration (BQSR) tool and GATK’s BaseRecalibrator and ApplyBQSR tools.

The search for variants was performed using GATK HaplotypeCaller, after which multilevel filtering was applied: low-quality variants were excluded (QUAL > 30, DP > 10), frequent variants were deleted (gnomAD_AF > 0.01) (https:// gnomad.broadinstitute.org). The variants were annotated using ANNOVAR (Wang K. et al., 2010) and the refGene, ClinVar, gnomAD, and dbNSFP databases. The analysis of rare pathogenic variants was conducted in accordance with the criteria of the American College of Medical Genetics and Genomics (Richards et al., 2015) and the clinical significance of the variants was assessed using ClinVar (https://www.ncbi. nlm.nih.gov/clinvar/). The predicted effect on the protein was evaluated using in silico tools such as SIFT (http://sift.jcvi. org), PolyPhen-2 (http://genetics.bwh.harvard.edu/wiki/pph2/ about) and PROVEAN (http://provean.jcvi.org/index.php).

## Results

A family with two patients, A. and I., born in 2015 and 2017, respectively, who have intellectual disabilities, consulted a psychiatrist at the Child Psychiatry Department of the V.M. Bekhterev National Research Medical Center located in St. Petersburg to clarify their diagnosis and select treatment. The patients were admitted to the hospital together with their mother to receive treatment.Patient A. An 8-year-old boy, born in 2015, Lezgin by nationality, has a family history of hereditary diseases. His younger brother has ID. He could sit since he was 7 months old, and walk since he was 1 year and 4 months old. Speech in the form of individual words began to appear around the age of 3.5. The perinatal period was burdened with complications, including a threat of pregnancy termination, anemia, and chronic fetal hypoxia. Urgent delivery was conducted by elective cesarean section at 41 weeks, birth weight was 3,500 grams (50th percentile), head circumference at birth was 35 cm (25th percentile), Apgar score was 7/7. During the neonatal period, the baby experienced prolonged jaundice and had feeding problems. He was also seen by an orthopedic specialist for diagnosis of pes valgus. Due to delays in speech development, the child was referred to a speech therapist. A speech delay of level III was identified, along with pseudobulbar dysarthria. The patient’s height is 122 cm, weight is 24 kg at the time of examination.By the time of treatment, his clinical picture showed signs of attention deficit hyperactivity disorder, aggression, tantrums, resistance to restrictions, lack of interest in studying, and sleep disturbance. He was consulted by a clinical psychologist for further evaluation. According to the results of the assessment, the psychologist identified an uneven intellectual development in the child, with a delay in verbal intelligence and difficulties with certain cognitive processes (attention, exhaustion of mental processes) of the organic type. During the work process, the boy required individual support due to his lack of self-organization skills and attention difficulties. In formal terms, according to the Wexler method, his verbal intellectual index (VIP) was 56 (in formal numerical terms corresponds to a mild level of underdevelopment), and his non-verbal intellectual index (NIP) was 94 (in formal numerical terms corresponds to the range of a low age norm).Magnetic resonance imaging (MRI) of the brain did not reveal any evidence of neoplastic or demyelinating processes or focal changes in the brain tissue.Video EEG monitoring of nighttime sleep revealed moderate changes in the bioelectric activity of the brain, with a predominance in the right frontocentral regions, increased excitability in deep structures at the diencephalic level and an increase in the phase of REM sleep II. However, no specific paroxysmal activity was recorded.Based on the clinical presentation and hospital exams, a speech delay was diagnosed in combination with intellectual disability and specific learning difficulties. The patient also had problems with activity and attention at the time of admission to the study. At the time of enrollment in the study, the patient was taking tiapride to manage excitability and aggressive behavior.Patient I. A 7-year-old boy, born in 2017, Lezgin by nationality, has a family history of hereditary diseases. His older brother has ID. He could sit since he was 7 months old, and walk since he was 1 year and 3 months old. Speech in the form of individual words began to appear around the age of 3. The perinatal period was burdened with complications, including a threat of pregnancy termination, anemia, and chronic fetal hypoxia. The mother gave birth by elective cesarean section on time, birth weight was 3,950 grams (50th–75th percentile), head circumference at birth was 37 cm (75th percentile), Apgar score was 7/7. The patient’s height is 115 cm, weight is 20.5 kg at the time of examination. The boy’s medical history includes pes valgus, chest wall deformities, enuresis and constipation. There were no visual or hearing impairments reported.Cognitive impairments and attention deficit hyperactivity disorder were prominent in the clinical presentation. According to the results of psychological assessment, delayed speech and insufficient development of verbal and logical components of intellectual activity were identified. In terms of formal numbers, based on the Wexler assessment, the productivity of intellectual functioning (for preschoolers, WPPSI) was at a moderate level of underdevelopment (VIP = 56) and the normative level (NIP = 100).MRI of the brain did not reveal any evidence of neoplastic or demyelinating processes or focal changes in the brain tissue.

Based on the clinical presentation and hospital exams, a mild intellectual disability with severe speech disorders, attention deficit hyperactivity disorder was diagnosed. At the time of enrollment in the study, the patient was receiving amitriptyline to manage attention disorders and hyperactivity.

The mother, born in 1988, Lezgin by nationality, has no known health problems. Her father experienced a severe heart attack and mother has an aggravated hernia. There were no medical or spontaneous abortions in the family’s reproductive history. She has an older sister and a younger brother, who also have a son and two daughters, respectively. The family pedigree is shown in the Figure A. The clinical manifestations identified in the siblings were not observed in other relatives in the family. Genitals, skin, and appendages are normal, and the skeleton is free of pathology. Vision and hearing are without pathology. Speech is not impaired, speech development is timely. The gait is normal. There was one episode of an affective phase with psychosis after childbirth.

**Fig. 1.**
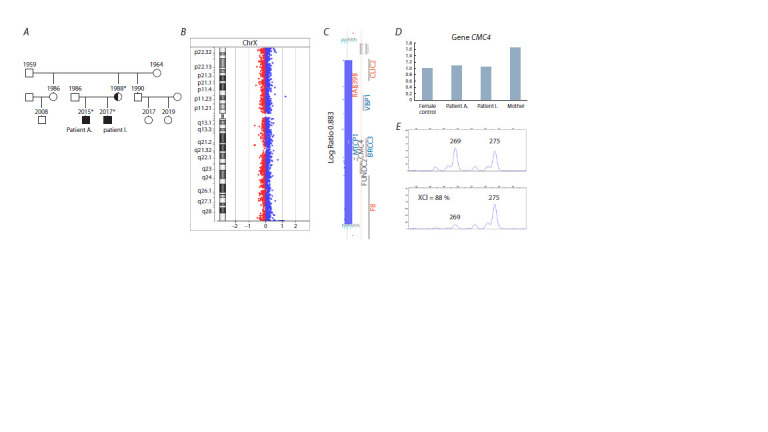
The results of the molecular cytogenetic analysis of the family. А – family history; * patients examined in this study. B – chromosome X profile from array-CGH of patient I. C – the blue bar indicates the region of duplication in the Xq28 chromosome of the patient and the genes within that region. D – Real-time PCR results (exon 3 of the CMC4 gene). The X axis – the control female DNA and the DNA of the examined individuals; the Y axis – multiple change in the number of DNA copies. E – analysis of the X-chromosome inactivation status in a carrier of the Xq28 duplication.

To clarify the causes of ID development in the family we performed a molecular cytogenetic study using array-based comparative genomic hybridization (see the Figure). We detected in both brothers a duplication of Xq28 on the X chromosome inherited from their healthy mother with skewed (88 %) X-chromosome inactivation. The size of the rearrangement is 439.6 kb. Eight genes are located in this region, including F8, MTCP1, BRCC3, VBP1, RAB39B, CLIC2, FUNDC2, and CMC4. The presence of CNV was confirmed in both siblings and their mother using real-time PCR.Whole-exome sequencing was performed for both siblings to exclude other potentially pathogenic variants in the coding regions of genes that could contribute to the development of the disease. After analyzing the data from the exome sequence, no pathogenic or likely pathogenic variants were found that could explain the observed clinical picture

## Discussion

Clinical observations show that intellectual disability is more prevalent in males than in females (Mental retardation in children, 2024). X-linked intellectual disability (XLID) is known to contribute to a significant proportion of ID in males, accounting for approximately 10–15 % of cases (Tolmacheva et al., 2022). To date, 114 different forms of XLID and 172 genes have been identified, variants in which can contribute to the development of the disorder (according to Greenwood Genetic Center, X-Linked Intellectual Disability) (Tolmacheva et al., 2025). Additionally, chromosomal microstructural rearrangements account for approximately 5 % of all cases of XLID (Bauters et al., 2008).We examined a family where two boys, aged 8 and 7, were diagnosed with mild intellectual disability and had a 439.6 kb duplication on chromosome Xq28 inherited from a healthy mother. Eight genes were located in this region, including F8, MTCP1, BRCC3, VBP1, RAB39B, CLIC2, FUNDC2, and CMC4

This chromosomal region overlaps with the region of Xq28 duplication syndrome (OMIM #300815). Xq28 duplication syndrome is a genetic condition linked to the X chromosome, causing ID and other neurodevelopmental issues. The syndrome is characterized by varying degrees of cognitive impairment, typically more pronounced in males. Affected individuals also experience a wide range of neurobehavioral abnormalities and facial dysmorphia (El-Hattab et al., 2011, 2015; Lannoy et al., 2013; Vanmarsenille et al., 2014; Voinova et al., 2015; Ballout et al., 2021). The main symptoms reported in patients with this syndrome are listed in the Table. In rare cases, duplication occurs de novo, but in most cases, affected boys inherit the distal duplication of the long arm of the X chromosome from their mothers. Heterozygous females did not show obvious clinical signs of the disease due to nonrandom X-chromosome inactivation (Amos-Landgraf et al., 2006; Lavrov et al., 2017; Tolmacheva et al., 2022). Sometimes mothers may have anxiety-depressive disorders, specific personality traits, speech difficulties, and seizures. We found skewed X-chromosome inactivation (88 %) in the mother with the Xq28 duplication, which may explain the lack of clinical symptoms of ID.

**Table 1.**
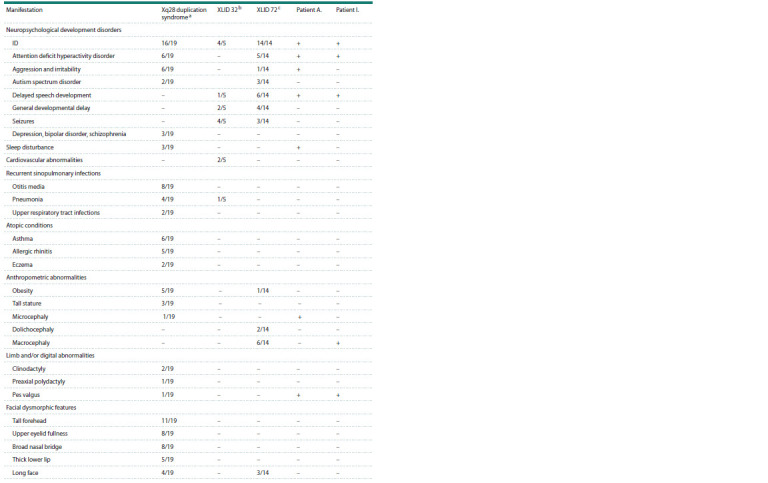
Comparison of patients clinical features with literature data Notе. a According to (El-Hattab et al., 2011, 2015; Lannoy et al., 2013; Vanmarsenille et al., 2014; Ballout et al., 2021). b According to (Witham et al., 2011; Takano et al., 2012). с According to (Russo et al., 2000; Giannandrea et al., 2010).

Xq28 region contains many sets of low-copy repeats (LCRs) in close proximity to each other, which render this region prone to non-allelic homologous recombination, which can lead to the formation of gametes with reciprocal microdeletions and microduplications (Vandewalle et al., 2009). The most frequently duplicated region includes the methyl-CpG-binding protein 2 (MECP2) gene, with a minimum duplication size of 0.2 million bp. Patients with MECP2 duplications have severe ID, incurable seizures and recurrent infections. Duplications in the telomeric regions, including the GDP 1 dissociation inhibitor (GDI1) gene and the RAS-associated RAB39B protein (RAB39B) gene, are independently associated with ID (Tolmacheva et al., 2022). It has been noted that the severity of clinical symptoms in patients with duplications of the GDI1 gene correlates with the number of copies of the gene (Vandewalle et al., 2009). It should be noted that in the clinical case we described, the duplicated region did not include the MECP2 and GDI1 genes, but included the RAB39B gene. The neurocognitive symptoms of the syndrome are speculated to be the result of an increased dosage of two genes located in the duplicated segment: RAB39B (OMIM #300271) and CLIC2 (OMIM #300138), due to the identification of both loci within the smallest region of the overlap between the duplicated segments in all affected individuals with Xq28 duplication syndrome (Andersen et al., 2014; El-Hattab et al., 2015).

CLIC2 encodes a unique transmembrane chloride channel found in cardiac and skeletal muscle cells. This channel interacts with the ryanodine receptor 2 (RyR2) in modulating calcium release from the sarcoplasmic reticulum within skeletal and cardiac myocytes (Board et al., 2004; Meng et al., 2009). Pathogenic missense variants in the CLIC2 gene are associated with a specific form of XLID (XLID 32, OMIM #300886) (Takano et al., 2012). The clinical manifestations of XLID 32 are presented in the Table. However, the effects of CLIC2 duplication remain uncertain, quantitative expression analysis suggests no significant dosage sensitivity (Vanmarsenille et al., 2014).

Another candidate gene that may contribute to the phenotype is RAB39B. The RAB39B gene encodes a member of the Rab protein family, which are small GTPases involved in intracellular signaling proteins that coordinate vesicle trafficking during a variety of cellular processes, including neuronal development and signaling (Mignogna et al., 2015). Pathogenic missense variants in the RAB39B gene are associated with a specific form of XLID (XLID 72 (OMIM #300271)) and Weisman syndrome (OMIM #311510). The clinical manifestations of XLID 72 are presented in the Table. Loss of function mutations in RAB39B have been recently linked to early onset of Parkinson’s disease (Wilson et al., 2014; Lesage et al., 2015). Four men with RAB39B duplication have been diagnosed with ID and behavioral disorders (Vanmarsenille et al., 2014). Additionally, overexpression of RAB39B in mouse primary hippocampal neurons demonstrated a significant reduction in neuronal branching and the number of synapses, resulting in impaired neuron development and synaptic dysfunction (Vanmarsenille et al., 2014). Neuronal overexpression of RAB39B impaired the recognition memory and the short-term working memory in mice and resulted in certain autism-like behaviors, including social novelty defect (Wang Z. et al., 2023).Therefore, the pathogenic role of this aberration is unclear due to limited information in databases. RAB39B is a dose-sensitive gene, with evidence of haploinsufficiency (ClinGen DS, https://search.clinicalgenome.org/kb/genedosage/ RAB39B). In this regard, based on the program for determining the pathogenic significance of CNVs (AutoCNV), the duplication of Xq28 containing this gene is assessed as a variant of uncertain clinical significance (with a score of 0). However, considering the segregation of inheritance in the family, item 5D should be selected in the AutoCNV program (“CNV is associated with a specific condition observed in the patient’s family”). This gives a score of 0.45. In a study, 88 % skewed X-chromosome inactivation pattern was observed in a mother, which added 0.65 points (Tolmacheva et al., 2025). Therefore, the overall score for this CNV is 1.1, which allows us to interpret this variant as pathogenic.

The clinical manifestations of the clinical case we studied were similar to those reported in the literature. We identified common symptoms, which are characteristic of Xq28, XLID 32, and XLID 72 duplication syndromes. These include intellectual disability, impaired speech development, and attention deficit hyperactivity disorder. The medical history of both boys includes pes valgus. Previously described as a rare manifestation of Xq28 duplication syndrome, we have observed those in both boys in our clinical case. The older brother has sleep disturbance, which is typical for patients with Xq28 duplication syndrome. At the same time, we have identified unique symptoms in the younger brother that have not been previously described, such as chest wall deformities and enuresis.

## Conclusion

By comparing the results of our molecular cytogenetic analysis with patient anamnesis data and information available in the literature, we have identified common clinical and phenotypic features (such as ID with other mental disorders and limb abnormalities) in boys with duplication of the Xq28 region, as well as in previously described patients with similar duplications, and in patients with ID, associated with variants in the CLIC2 (XLID 32) and RAB39B (XLID 72) genes. Whole-exome sequencing did not reveal pathogenic and likely pathogenic variants associated with neurodevelopment disorders. The size of the rearrangement is 439.6 kb. Eight genes are located in this region, including F8, MTCP1, BRCC3, VBP1, RAB39B, CLIC2, FUNDC2, and CMC4. For the detected CNV, the total score according to the ACMG algorithm considering the X-chromosome inactivation status was 1.1. Based on the overall results, this variant may be interpreted as pathogenic, which may lead to clinical symptoms in patients. Based on the analysis of clinical cases reported in the literature, it is possible to assume that cognitive impairments may be associated with an increased expression of the RAB39B gene due to changes in the number of copies of this region.

## Conflict of interest

The authors declare no conflict of interest.
